# An aquatic environmental DNA filtration system to maximize recovery potential and promote filtration approach standardization

**DOI:** 10.7717/peerj.15360

**Published:** 2023-07-12

**Authors:** Hayley M. DeHart, Mark T. Gasser, Jarret Dixon, Peter Thielen

**Affiliations:** 1 Research and Exploratory Development Department, The Johns Hopkins University, Laurel, MD, United States of America; 2 Research and Exploratory Development Department, The Johns Hopkins University Applied Physics Laboratory, Laurel, MD, United States of America; 3 Force Projection Sector, The Johns Hopkins University Applied Physics Laboratory, Laurel, MD, United States of America

**Keywords:** Environmental DNA, eDNA, Field analysis, Biodiversity, Invasive species

## Abstract

**Background:**

Aquatic environmental DNA (eDNA) has emerged as a promising approach to identify organisms in freshwater and marine environments. While the recovery of eDNA from water most commonly involves capture of biological debris on a filter matrix, practitioners are yet to converge on standardized approaches for filtration, particularly in the field. This lack of standardization has resulted in inconsistent handling of samples following collection, limiting interpretation of results across studies and burdening groups with inconvenient storage and transport logistics that may compromise eDNA integrity.

**Methods:**

A simple to assemble and low-cost ($350 USD) water filtration system is demonstrated that can be used in field and laboratory settings to reduce time between sample acquisition and eDNA filtration, maximizing eDNA sample recovery. Quantitative PCR is used to show the utility of the platform for laboratory and marine eDNA analysis.

**Results:**

The resulting eDNA collection system is easily transported in a rugged water-resistant case, operates for more than eight hours on a 12-volt lead-acid battery, and has an unobstructed filtration rate of 150.05 ± 7.01 mL/min and 151.70 ± 6.72 mL/min with 0.22 µm and 0.45 µm Sterivex filters, respectively. We show that immediate sample filtration increases eDNA recovery in the laboratory, and demonstrate collections in aquaria and marine environments. We anticipate that providing easy to obtain, open hardware designs for eDNA sample collection will increase standardization of aquatic eDNA collection methods and improve cross-study comparisons.

## Introduction

Environmental DNA (eDNA) has emerged as a powerful tool for the characterization of aquatic biological communities. eDNA metabarcoding technology adoption is being driven by advances in low-cost DNA sequencing and improved analysis methods that capture genetic diversity from both cataloged and previously undescribed species (Chavez et al., 2021). Aquatic eDNA research is rapidly developing, and the published literature contains many approaches for sample collection and processing (Helbing & Hobbs, 2019). This rapid expansion of eDNA methods makes it challenging for researchers to understand sampling best practices and establish new eDNA workflows of their own.

Meta-analysis efforts have aimed to identify common practices and identify standard operating procedures for intermediate sample preservation, ideal filter matrices, and DNA isolation prior to genetic analysis (Miya et al., 2016). A common set of collection, process, and analysis approaches has recently been proposed to establish standards of practice that can be adopted by the larger research community, with the aim of enabling comparative research across the expanding field of molecular ecology (Sepulveda et al., 2020; Minamoto et al., 2020). Filtration-based methods are the most commonly used method for eDNA collection and concentration from water samples (Tsuji et al., 2019). However, considerable variation remains in collection approaches due to the wide range of eDNA applications, differences in individual project logistics, and the availability or accessibility of filtration equipment (Nagler et al., 2022). This variation has led to the method and time to filtration often being incompletely described or omitted in the literature.

Common manual approaches for sample filtration involve forcing water through a filter using a large-volume (50–60 mL) syringe (Mächler et al., 2016; Sigsgaard et al., 2017; Minamoto et al., 2020) or the combination of a hand-pump and vacuum flask (Baldigo et al., 2017; Kamoroff & Goldberg, 2018). This process requires significant manual force, and as a result, is generally limited to less than 0.5L collection volume. Alternatively, filtration is performed using a laboratory-grade vacuum or peristaltic pump (Walsh, Zaikova & Hallam, 2009; Miya et al., 2016; Djurhuus et al., 2017), which typically requires researchers in the field to transport collected water samples to a secondary location suitable for filtering. Field-ready integrated systems are relatively new to the market, and have shown promise for collection, preservation, extraction, and targeted aquatic invasive species detection (Thomas et al., 2018; Thomas et al., 2020). However, the initial cost and recommended use of proprietary filters may not address all potential deployment needs. Similarly, Formel et al. (2021) developed an automated sampling device capable of sample collection and subsequent preservation, but this device requires access to fabrication facilities capable of 3D printing and microelectronics assembly.

Several studies have identified technical advantages of enclosed Sterivex™ filter cartridges for aquatic eDNA collection (Miya et al., 2016; Spens et al., 2017; Tsuji et al., 2019; Ushio, 2019; Takahashi et al., 2020). These filters are individually packaged, sterile, and lightweight—all features that make them ideal for transport into field collection locations. Despite the advantages offered by Sterivex™ filtration cartridges, their use for eDNA collection can be challenging due to pressures required (1–3.1 bar, or 15–45 psi) for manual collection with large-volume syringes, or mechanical collections using vacuum or peristaltic pump systems. Significant pressure is required to pull or push liquids through the filtration matrix as it becomes clogged with particulate matter. Vacuum- or pressure-driven systems require robust laboratory equipment to generate sufficient force, which are often large and require a standard 110V/220V power source. Additionally, our group has found that standard peristaltic or vacuum pumps can produce excessive pressure or suction that causes collapse of tubing or separation of the filter body from the pump as the membrane becomes clogged and internal pressure increases. Similarly, standard laboratory vacuum and peristaltic pumps are costly and may generate insufficient pressure for filtration of samples when using a manifold system to increase the number of samples being filtered.

To address these issues, we have designed a field-portable pump to minimize the need for water transfer to a traditional laboratory for filtration. This pump reduces the time from sample collection to filtration by hours or even days, depending on sampling locations and access to laboratory pumps, and therefore the potential for degradation or unintentional alteration of sample integrity due to increased or decreased biological activity within a water sample. Once samples are filtered, they can be quickly preserved with chemical reagents to eliminate the need for cold-chain storage. We provide detailed pump assembly instructions (Supplemental Document), which takes less than 30 min to assemble at a cost of ∼$350 USD. The resulting design incorporates three key features that address several gaps in the existing aquatic eDNA literature:

1. It is rugged, lightweight (∼5 lb or 2.2 kg—equivalent to ∼2 liters of water), and uses a rechargeable battery with capacity for at least 30L of water per battery charge. In addition, the secured and small footprint case allows for packed transport in a backpack or checked luggage during airline travel.
2. The design utilizes a Sterivex™ filtration cartridge (MilliporeSigma, Burlington, MA, USA) under negative pressure, eliminating potential for contamination from fouled tubing or other processing equipment. However, no modifications to the core device are necessary to use other filter types. Compatibility with other filtration systems can be attained simply by choosing the tubing and couplers needed for connection.
3. A minimal and robust design that can be quickly modified, repaired, and improved with ease-of-operation makes this device interchangeable whether sampling in the field or laboratory.

The system described was tested for flow rates, portability, as well and total and targeted eDNA recovery using Sterivex™ in several common eDNA sampling efforts, including controlled laboratory experiments, local transport and land-based collection, and field-based collection on a research vessel. Through use of a portable, easy to assemble eDNA filtration system, we aim to reduce the dependency on expensive, non-portable systems that require transportation of large water volumes.

## Materials and Methods

**Figure 1:**
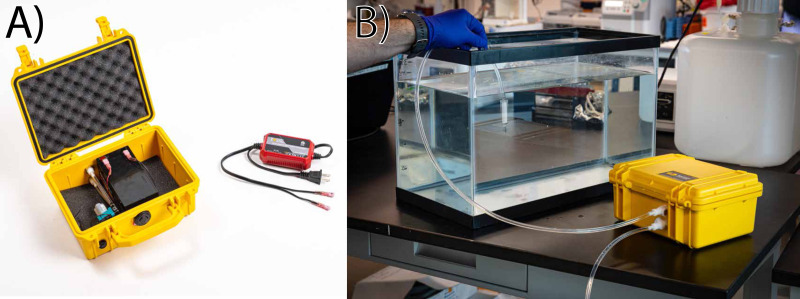
Assembled pump and illustrative operation. The entire eDNA filtration setup (A) is housed in a pelican case, which contains the pump, lead–acid battery, and a trickle charger modified to connect to the battery terminals. During operation (B) tubing is connected to quick-release fittings, and a sterivex or similar filter housing is attached with compression mating between the filter and tubing. This pre-filtration approach reduces contamination potential from the tubing, and also prolongs the life of the pump by reducing or eliminating entry of particulate matter.

The eDNA collection system operates as a negative pressure filtration device in which tubing attaches to the male port of a Sterivex™ or other filtration cartridge (Fig. 1 and Supporting Information). As described, the interior tubing size produces a pressure-fit connection with a Sterivex™ filter cartridge. Adaptations to the tubing and connectors could be adapted for use with other filter types, such as syringe filters or reusable Swinnex disc-filter holders, using alternative tubing sizes, types, or adapters. Designs referenced in this manuscript are licensed under CERN Open Hardware License v2—Permissive (CERN-OHL-P v2).

Assembly of the pump is divided into four steps, which are outlined in detail with images in the supplemental document. First, the enclosure is altered with three 12.7 mm (0.5 inch) holes to accept quick-disconnect tubing and a switch. Second, the pump is attached to quick-disconnect couplers and wired to the switch and battery. Third, sampling tubing is modified to add quick disconnect couplers. Last, the trickle-battery charger is modified to interface with the lead–acid battery terminals. The entire unit can be assembled in approximately 30 min, and the total cost of materials for one unit is approximately $350 USD at the time of publication (Table 1).

**Table 1:** Bill of materials for single pump assembly.

Component	Supplier (Part Number)	Price	Quantity	Total Cost
Pelican Case 1150	Pelican (1150)	$42.95	1	$42.95
Firm Polyethylene tubing for Air and Water (0.125″ID, 0.25″OD; 25 ft)	McMaster-Carr (5648K74)	$19.00	1	$19.00
Quick-disconnect Receptacle	McMaster-Carr (5012K19)	$6.37	2	$12.74
Quick-disconnect Plug	McMaster-Carr (5012K53)	$9.49	2	$18.98
12V Custom Diaphragm Pump (0.23L/min)	KNF (PML17183-FF20 IP40)	$163.00	1	$163.00
Battery	Expert Power (EXP1213)	$19.99	1	$19.99
Automatic Trickle Battery Charger 12V 1000mA	Foval (BC01B-1)	$20.98	1	$20.98
Waterproof Switch, SPST with wire leads	Mouser (IPR1FAD2)	$34.57	1	$34.57
Molex Perma-Seal butt splice Connectors 18-22 AWG (50-pack)	Mouser (19164-0811)	$20.12	1	$20.12
Molex Perma-Seal Female Terminals 18-22 AWG	Mouser (19164-0017)	$1.03	2	$2.06
Total Pump Cost:				**$354.39**
*All pricing as of May 2022				

Flow rate characterization was performed with a fully charged battery and a 0.5-meter inlet tube. 3.0 L of synthetic sea water from a marine invertebrate housing tank were filtered with 0.22 µm and 0.45 µm filters, and the volume of water filter was recorded each minute until total the volume was filtered. We recognize that synthetic seawater filtration represents an ideal scenario for filtering, without physical and biological materials that will slow filtration rates and clog pumps, but present maximum filtration rates in seawater for baseline estimations of flow rate.

### Laboratory experiments

Pumps were utilized for three separate experiments to characterize eDNA quantities and eDNA degradation rates that may occur if time-to-filtration is increased using our pump system. In the laboratory, 40 blue mussels from our research stock were removed, rinsed with DI water and placed in 15 L of artificial seawater with continuous aeration and kept overnight at room temperature (∼20 °C) without feeding. The tank housing was cleaned with detergent, exposed to 10% bleach solution for 10 min and thoroughly rinsed with DI water prior to adding seawater and mussels. All mussels were removed the following morning and 6 L of water were collected from the tank. The water was assumed to be homogeneous after mixing and distributed equally to 12 samples of 500 mL. An additional three samples of 500 mL of artificial control seawater were taken from the tank prior to the addition of mussels. All sampling containers were decontaminated by 10% bleach soak for 30min and rinsed with DI water prior to sampling.

In replicates of four, water samples (500 mL) were filtered using our device at 0, 4, and 16 h. The time after removing the mussels from the tank was defined as time 0, and water samples were stored at 4 °C until filtration at 4- and 16-h timepoints. One (500 mL) control sample was filtered at each time point. DNA was captured using a Sterivex™ cartridge filter (0.45 µM pore size, PVDF membrane, MilliporeSigma). All residual water was removed from the cartridge and the filters were immediately placed into an −80 °C freezer until DNA extraction. Quantitative PCR was performed using species-specific nuclear markers (Hamer et al., 2010) and mitochondrial COI markers for marine invertebrates (Geller et al., 2013).

### Aquarium and field collections

eDNA was collected using the filtration system at the National Aquarium (Baltimore, MD, USA) in December 2021 and during a field exercise in Monterey Bay (CA, USA) in April 2022. Samples were collected from the Atlantic bottlenose dolphin (*Tursiops truncatus*) exhibit at the National Aquarium, and from areas with marine mammal sightings in Monterey Bay. One liter was filtered at each site, DNA was captured using a Sterivex™ cartridge filter (0.45 µM pore size, PVDF membrane, MilliporeSigma, Burlington, MA, USA), and samples were filtered on-site within one hour of collection. Immediately after filtration, filter cartridges were filled with ∼0.8 mL of Buffer ATL (Qiagen, Hilden, Germany) and kept at −20 °C until DNA extraction. Buffer ATL was chosen as a storage and preservation medium in which the eDNA would be unlikely to degrade outside of freezer conditions.

### DNA extraction and quantitative PCR

eDNA was isolated from Sterivex™ cartridges using a modified Qiagen DNeasy Blood and Tissue kit (Qiagen, Hilden, Germany) protocol. Briefly, 720 µL of ATL buffer and 80 µL of Proteinase K were added directly to the filter in the cartridge, and incubated at 56 °C in a rotating incubator for 2–24 h. After incubation, all liquid was transferred from the cartridge and an equal volume of buffer AL and cold (4 °C) 200 proof molecular biology grade ethanol were added before continuing with manufacturer’s protocol. All eDNA extractions were stored at −20 °C until quantitative PCR (qPCR) analysis.

To evaluate mitochondrial copy-number in aquaria and field-collected samples, we synthesized a plasmid containing a conserved marine vertebrate mitochondrial sequence (MarVer) that incorporates ∼2200 bp spanning mitochondrial 12S and 16S ribosomal RNA genes (Valsecchi et al., 2020). This plasmid was developed from the Caspian seal (*Phoca caspica*) sequence and was conserved across the MarVer primer regions described. Plasmid DNA was quantitated using a Qubit fluorometer (Thermo Fisher Scientific, Waltham, MA, USA), and then serially diluted to copy numbers spanning from 10^8^ to 10^1^ copies/rxn. These reactions were scaled to create a standard curve against which the unknown samples were compared to and quantitated.

PCR reactions were run using a 2x Fast SYBR qPCR Master Mix (Thermo Fisher Scientific, Waltham, MA, USA) on an Aria Mx Real-Time PCR System (Agilent, Santa Clara, CA, USA). For laboratory experiments, reactions were 20 µL in volume and each included 10 µL of 2x Master Mix, 0.5 µL forward and reverse primers at 10 µM concentration (Table 2), 6.5 µL nuclease-free water, and 2.5 µL of sample template. Cycling conditions consisted of initial denaturation at 94 °C for 10 min, followed by 40 cycles of 94 °C for 15 s and 60 °C for 60s. Aquarium and field samples were amplified for the MarVer1 (12S) and MarVer3 (16S) regions using marine-mammal specific primers (Valsecchi et al., 2020). Reactions included 10 µL of 2X Master Mix, 1 µL forward and reverse primer at 10 µM, 7 µL nuclease-free water, and 2 µL of sample template. Cycling conditions consisted of UDG activation at 50 °C for 2 min, initial denaturation at 95 °C for 2 min, followed by 40 cycles of 95 °C for 15 s, 58 °C for 15 s, and 72 °C for 1min. All reactions were performed in triplicate and included a negative PCR control.

**Table 2:** Primers used in qPCR experiments.

**Name**	**Marker Location**	**Specificity**	**Forward Primer (5′-3′)**	**Reverse Primer (5′-3′)**	**Source**
jgLCO1490/ jgHCO2198	Mitochondrial Cytochrome Oxidase Subunit I	Invertebrate	TITCIACIAAYCAYAARGAYATTGG	TAIACYTCIGGRTGICCRAARAAYCA	Geller et al. (2013)
Me 15/16	Nuclear Locus	*Mytilus edulis*	CCAGTATACAAACCTGTGAAGA	GTTGTCTTAATAGGTTTGTAAGA	Wilson et al. (2018)
MarVer1	Mitochondrial 12S Ribosomal RNA	Marine Mammal	CGTGCCAGCCACCGCG	GGGTATCTAATCCCAGTTTG	Valsecchi et al. (2020)
MarVer3	Mitochondrial 16S Ribosomal RNA	Marine Mammal	AGACGAGAAGACCCTATG	GGATTGCGCTGTTATCCC	Valsecchi et al. (2020)

## Results

### System filtration performance

Filtration rates using 0.22 µm and 0.45 µm Sterivex filters were observed at 150.05 ± 7.01 mL/min and 151.70 ± 6.72 mL/min, respectively. This allows for 1L sample filtration in approximately 6.5 min using common filter pore sizes. Total DNA extraction yields ranged across lab, aquarium, and field samples, with lab samples averaging 977 ng (±85 ng S.E.), aquarium samples averaging 745 ng (±24 ng S.E.), and field samples averaging 1,764 ng (±330 ng S.E.).

### Laboratory experiments

**Figure 2:**
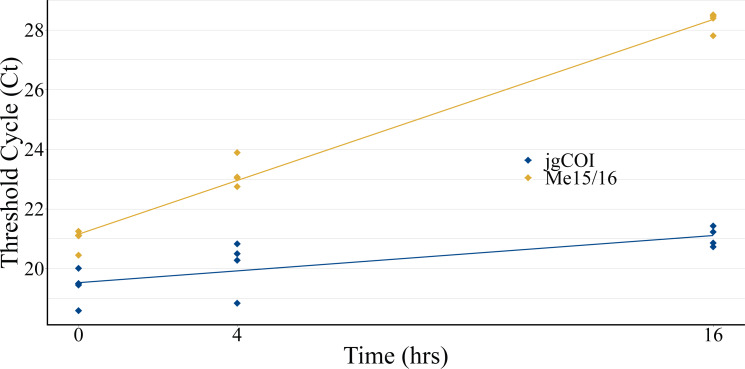
Water collection timecourse to assess eDNA stability. eDNA samples were collected from a single tank of water formerly containing mussels (*Mytilus edulis*) over a 16-hour period to assess qPCR cycle threshold value stability. Mitochondria-specific markers (blue; jgCOI) displayed similar values over the period, while a single-species nuclear marker (orange; Me15/16) demonstrated reduced PCR product formation *via* higher Ct values.

Experiments to observe eDNA collection efficiency over time displayed increased Ct values for both blue mussel-specific nuclear markers and universal marine invertebrate mitochondrial markers, indicating lower DNA recovery over time (Fig. 2). Species-specific nuclear marker values (Me15/16) increased cycle threshold (Ct) from an average of 20.98 at T0 to 28.30 at T16. Universal marine invertebrate mitochondrial COI marker values (jgCOI) increased from an average Ct of 19.39 at T0 to 21.06 at T16.

### Aquarium and field collections

**Figure 3:**
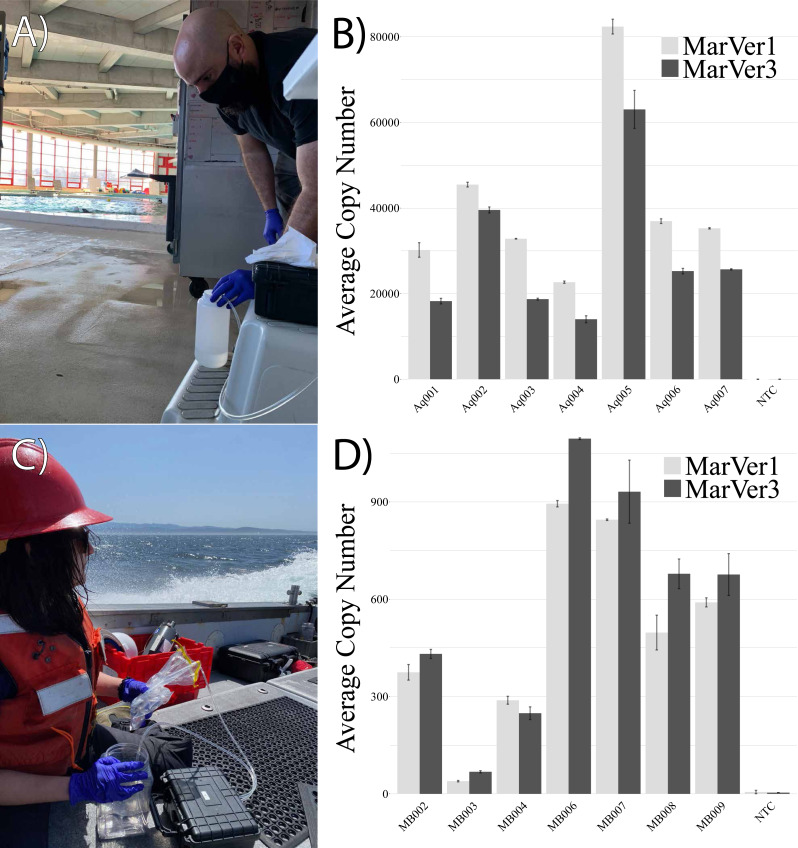
eDNA collection in field-based environments. Examples of eDNA sample collection at the National Aquarium bottlenose dolphin habitat in Baltimore, Maryland (A: photo credit Maddison Harman) and subsequent quantitation of eDNA using mitochondrial 12s MarVer1 and 16s MarVer3 primer sets to assess eDNA copy number (B). In open-ocean settings, collections performed while underway in a small boat between sampling locations in Monterey Bay, California (C: photo credit Hayley DeHart) and assessment of marine vertebrate (12s and 16s mtDNA) eDNA copy number in field-collected samples.

The MarVer qPCR assay was validated using our synthesized DNA standard at 100.1% and 97.8% efficiency for MarVer1 and MarVer3, respectively. Aquarium samples displayed mtDNA copy numbers within an order of magnitude of each other, varying between 10,000–80,000 copies per reaction, with an average of 40,831 (±7,404 S.E.) copies per reaction for MarVer1, and 29,224 (±6,439 S.E.) copies per reaction for MarVer3. These values were well within the quantifiable range of the qPCR assay (Fig. 3). Samples from Monterey Bay displayed lower copy number for MarVer1 and MarVer3 when compared to aquaria-collected samples, with all samples exhibiting fewer than 1,000 copies per reaction. MarVer1 displayed average copy number of 504 (±115 S.E.), while MarVer3 had an average copy number of 590 (±138 S.E.). The discrepancies in copy numbers between aquarium samples and field samples, despite high overall DNA yield, are potentially due to salt-based PCR inhibition and concentrations of marine vertebrate eDNA in the respective aquarium and natural environment sampling sources, and are unlikely due to mechanical collection differences between sites.

## Discussion

We have demonstrated a field-ready, rugged, and affordable aquatic eDNA collection system that produces high-quality eDNA samples suitable for qPCR-based detection and next-generation sequencing. Results indicate high eDNA yields from laboratory-based experiments, controlled aquarium systems, and field samples. The system can be easily assembled with minimal effort, training, and equipment, and can also be easily modified and repaired using parts available at hardware stores. For the reasons listed above, our group has increasingly used this system in remote-field environments to enable in-place eDNA collections.

There is conflicting evidence in the rate of eDNA degradation after it is shed from its host in the literature, including suggestions that eDNA degrades exponentially with time (Thomsen et al., 2012; Yamanaka et al., 2016), and that the time and method of storage prior to filtration affect sample detection (Takahara, Minamoto & Doi, 2015; Yamanaka et al., 2016; Hinlo et al., 2017; Curtis, Larson & Davis, 2020). Curtis, Larson & Davis (2020) described a significant decrease in observed operational taxonomic units (OTU) diversity from samples that had been stored for as little as one-hour post-collection, and Takahashi et al. (2023) observe an increase in field studies with on-site filtration in an attempt to preserve intact DNA. While our laboratory experiment Ct value increases are suggestive of degradation in samples that occurs between collection and filtration, discrepancies in nuclear *versus* mitochondrial markers could alternatively be attributed to differential decay rates between nuclear and mitochondrial DNA, or PCR efficiencies between the two primer sets. Additionally, our degradation experiment was limited to a single-species assay with few replicates across time points, and further experimentation would be needed to effectively assess degradation in water sources across time compared to on-filter degradation. However, our preliminary results, along with many others that have aimed to identify eDNA degradation rates (Spens et al., 2017; Friebertshauser et al., 2019), support the importance of immediate filtration and chemical preservation of samples collected in the field.

While laboratory-grade pumps may provide faster flow rates and increased collection volumes, we have observed that use of broadly available Sterivex™ or disc filters in the field increases the overall number of sample collection opportunities. With the myriad of water conditions and compositions being sampled by aquatic eDNA researchers, it is important to note that no eDNA collection method offers a solution to filter clogging due to organic matter, and high organic content samples would potentially benefit from pre-filtering samples to reduce clogging and facilitate larger sampling volumes.

Field-based sampling efforts showed effective collection of eukaryotic mitochondria using the pump and 0.45 µM filters, evidenced by successful qPCR of mitochondrial markers in aquarium and field samples. Higher copy numbers of MarVer1 and MarVer3 were found in aquarium samples as opposed to field samples from Monterey, which is expected as the concentration of marine mammals in the captive aquarium environment are far higher than field samples. Furthermore, the process of sampling and filtering on-site reduced the need to transport water samples back to a laboratory location for filtering, reducing risk of sample contamination or degradation during transport. The ability to easily transport eDNA filtration capabilities rather than transport heavy water samples greatly reduces the logistical burden for aquatic eDNA collections.

Our eDNA collection system has been distributed to multiple collaborators undertaking fieldwork ranging from polar oceans to tropical environments. Their feedback has informed refinement of the platform to reduce complexity and enable field-based repairs when necessary. As an open-hardware concept, future versions of this hand-portable filtration system could leverage higher-flow pumps, integrated flow-sensors, and battery management to improve usability. Currently, these pumps are being utilized on Lindblad Expedition ships, National Geographic Pristine Seas ships, and the Smithsonian National Museum of Natural History research program. Input from collaborators has guided design considerations, and we have expanded on the design described in this manuscript to improve field-reliability and throughput.

## Conclusions

We developed a low-cost, portable filtration system for aquatic eDNA collection that may be built and used with very little prior experience in hardware construction or eDNA sampling. By incorporating point of collection filtration into eDNA sampling workflows, we anticipate reduced dependencies on technical personnel for ecological inventories, increasing the consistency of biological survey data and potentially identifying cryptic organisms that are otherwise challenging to detect. Moving forward, we advocate for recording the period of time between initial sampling and filtration of aquatic eDNA samples.

##  Supplemental Information

10.7717/peerj.15360/supp-1 Data S1 Datasets and associated R code used to generate figures

10.7717/peerj.15360/supp-2 Supplemental Information 2 Assembly Instructions for eDNA Pump

## Additional Information and Declarations

### Data Availability

The following information was supplied regarding data availability: The raw data and associated R code are available in the Supplementary File.
