# Supplementary material for: An aquatic environmental DNA filtration system to maximize recovery potential and promote filtration approach standardization
Source: PeerJ. 2023 Jul 12;11:e15360. doi: 10.7717/peerj.15360 (PMC10349554; doi:10.7717/peerj.15360)
Supplement: Supplemental Information 2 [file peerj-11-15360-s002.pdf]

An Aquatic Environmental DNA Filtration System to Maximize Recovery Potential and Promote Filtration Approach Standardization

Hayley M. DeHart [1*], Mark Gasser[1*], Jarret Dixon[1], Peter Thielen[1]

[1]Johns Hopkins University Applied Physics Laboratory, Laurel, Maryland, USA

Supporting Information – Assembly of pump

Step 1. Enclosure Modification

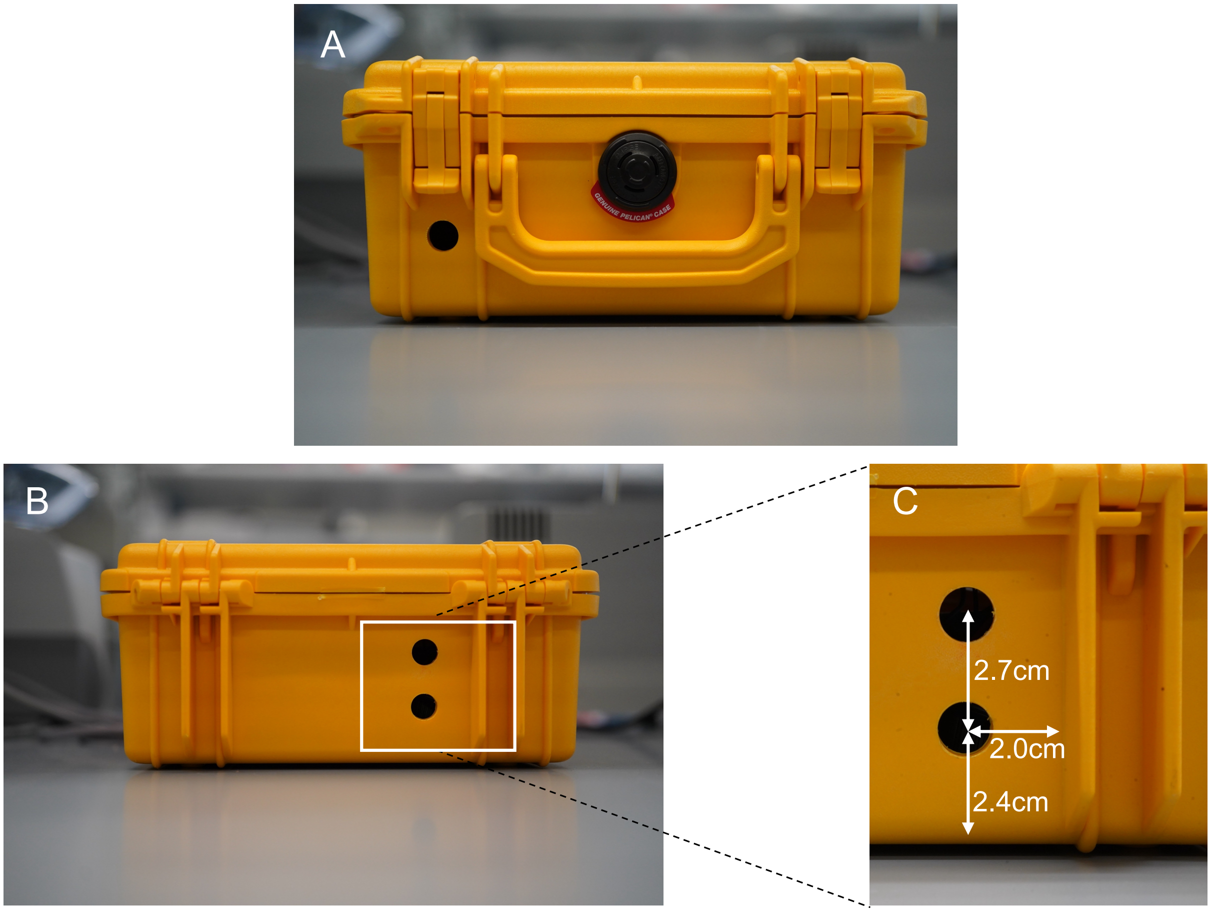

Figure 1: Case modification for quick-connect tubing and pump switch components
Three 13mm (0.5in) holes are made with a standard power drill in the sides and top of the case to attach the panel-mount tubing quick disconnects and switch. A single hole is drilled on the top of the enclosure to accommodate the water-resistant switch (Panel A). Holes for quick disconnect fittings are drilled on the side of the enclosure (Panel B, white inset) with spacing to accommodate the internal dimensions of the pump (Panel C). The switch should be near the enclosure clasp, without being obstructed by the clasp mechanism, to avoid unintentional engagement during transport.

Step 2: Assembly of internal pump components

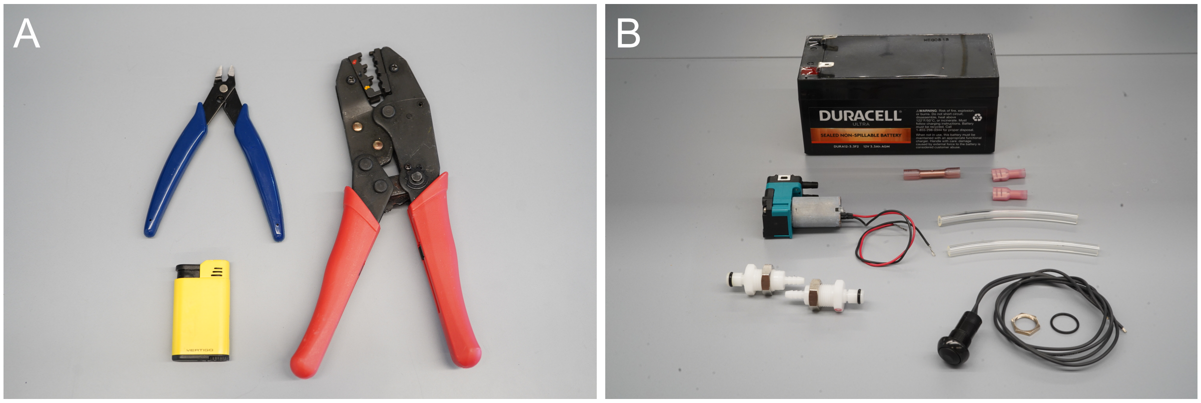

Figure 2: Tools and materials for pump assembly
Internal component assembly requires three tools (Panel A, clockwise from top left): a wire-cutter for trimming attached wires to appropriate lengths, a crimping tool for making connections between the pump, switch, and battery, and a heat source to shrink the tubing on the connectors. There are eight components in the internal assembly, including the pump, 12V battery, terminal connectors (2), switch, butt-splice connector, and tubing quick disconnects (2) (Panel B, clockwise from top left). Two short lengths of tubing are used to connect the quick disconnects and pump (not pictured).

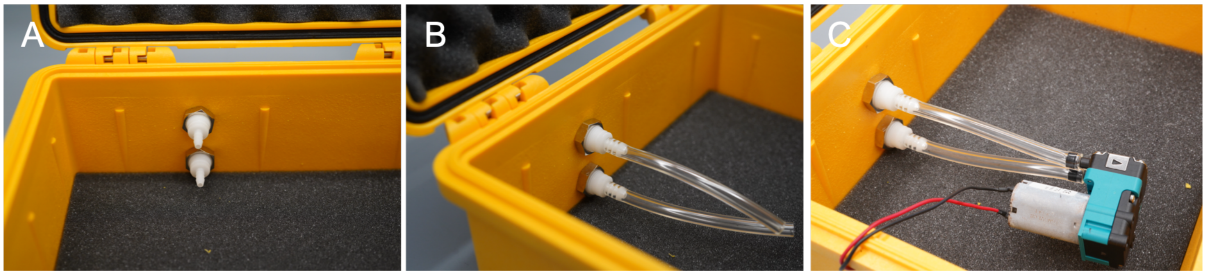

Figure 3: Connection of pump to quick-disconnect attachments
Quick disconnect couplings are inserted with the threaded portion to the inside of the enclosure, then hand-tightened with the nuts (Panel A). Short lengths of tubing (approximately 5cm) are heated briefly with a lighter to make the material more malleable, then pressed onto quick disconnect barb (Panel B). The tubing is then heated from within the case and fitted to the pump inlet/outlet barbs (Panel C).

Notes on waterproofing: Drilling holes in the enclosure negates the standard case weather sealing. Additional waterproofing can be achieved by adding o-rings or silicone sealant to the quick-disconnect fittings. The switch comes fitted with an o-ring, which should be used during installation, and waterproofing can additionally be improved by adding silicone sealant.

Option 1**:** 0.5in rubber o-rings can be added between the quick disconnect fitting and the exterior of the enclosure.
Recommended product: EDPM Water and Steam resistant o-rings; McMaster Carr part number 9557K443.

Option 2: Silicone adhesive can be added between the quick-disconnect and enclosure. Insert the threaded portion of the quick disconnect through the drilled holes, and loosely attach the nut prior to adding a bead of silicone adhesive to the exterior threading.
Recommended product: 3M Marine Grade Silicone Sealant, part number 8019.

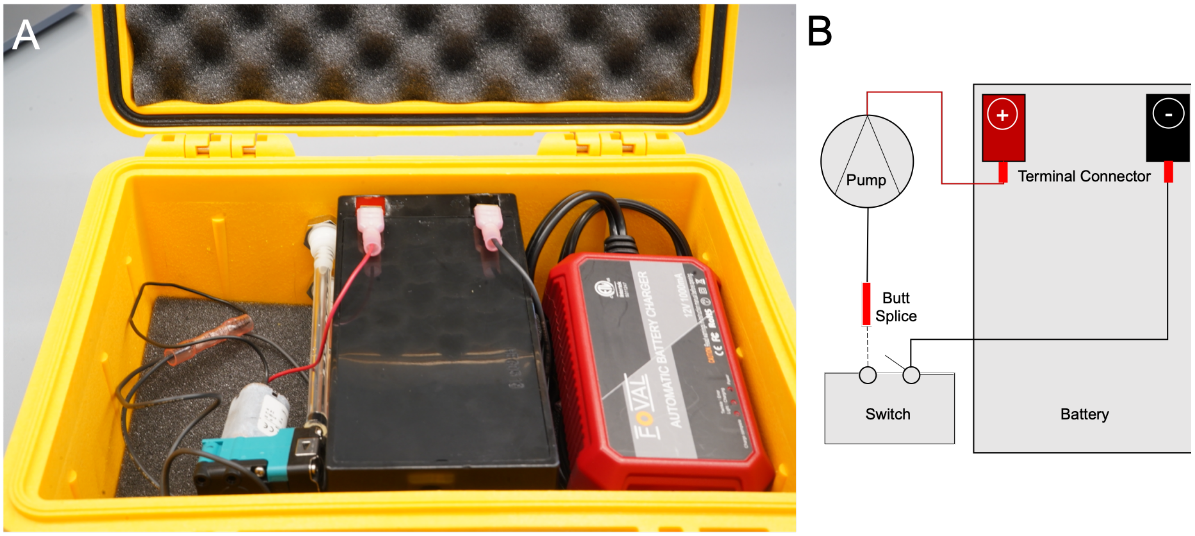

Figure 4: Pump internal components and wiring diagram
The completed pump assembly, including the battery charger (Panel A). All components and connections (salmon) for each electrical component, including the pump, switch, and battery (grey), as well as the pre-attached wiring from the pump and switch (Panel B).

Step 3: Assembling Quick-Disconnect Tubing

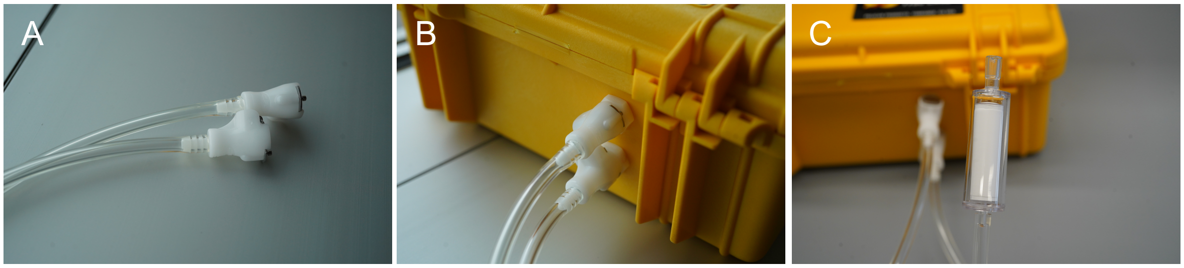

Figure 5: Addition of quick disconnects to tubing for pump and Sterivex filter connection
Quick disconnect couplers are added to one end of each piece of tubing, using a barbed attachment (Panel A). These attachments fit the male fittings connected to the outlet (Panel B, left connection) and inlet (Panel B, right connection). Sterivex™ filters connect directly to the tubing by resistance without the need for the luer lock fitting (Panel C).

Step 4: Modification of the Battery Charger

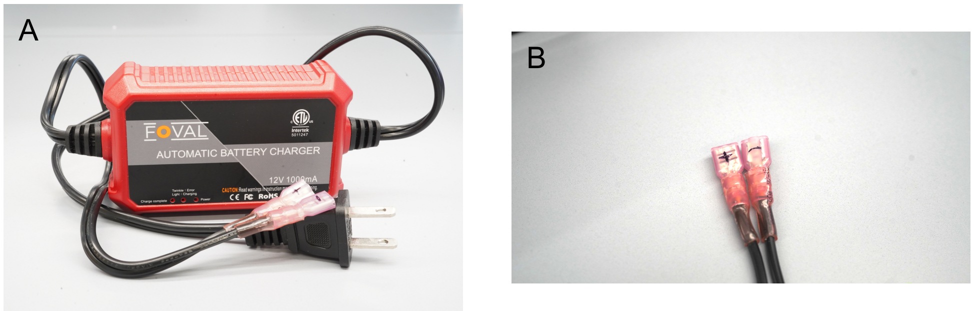

Figure 6: Modification of the battery charging device
Recharging lead-acid batteries is performed with an automatic battery charger rated for 12V batteries. These systems can be used in stock form, or modified with terminal connectors to securely connect to the battery (Figure A). The ends should be marked appropriately with "+" and "-" symbols to avoid incorrect polarity (Figure B). The negative wire should have lettering (printed or embossed) on it, but may vary by lot or manufacturer and should be confirmed.

Notes on battery charging: There are many different automatic battery chargers available from Amazon and other suppliers. The Foval charger specified is 12V / 1000mA, and has overcharging protection. If a standard 12V power supply (e.g. not a proper charging device) is connected to the battery, there is a possibility that it will be damaged, explode, or catch on fire.
